# An Outbreak of Equine Herpesvirus-4 in an Ecological Donkey Milk Farm in Romania

**DOI:** 10.3390/vaccines10030468

**Published:** 2022-03-18

**Authors:** Alexandra Mureşan, Cosmin Mureşan, Madalina Siteavu, Electra Avram, Diana Bochynska, Marian Taulescu

**Affiliations:** 1 Department of Internal Medicine, Faculty of Veterinary Medicine, University of Agricultural Sciences and Veterinary Medicine of Cluj-Napoca, 400372 Cluj-Napoca, Romania; 2 Department of Surgery, Anesthesia and Intensive Care, Faculty of Veterinary Medicine, University of Agricultural Sciences and Veterinary Medicine of Cluj-Napoca, 400372 Cluj-Napoca, Romania; cosmin.muresan@usamvcluj.ro; 3 Synevovet Laboratory, B-dul Pache Protopopescu, Nr. 81, 021408 Bucuresti, Romania; madalina.siteavu@synevovet.ro (M.S.); electra.avram@synevovet.ro (E.A.); marian.taulescu@usamvcluj.ro (M.T.); 4 Department of Pathology and Forensic Medicine, Faculty of Veterinary Medicine, University of Agronomic Sciences and Veterinary Medicine, 050097 Bucharest, Romania; 5 Department of Anatomical Pathology, Faculty of Veterinary Medicine, University of Agricultural Sciences and Veterinary Medicine of Cluj-Napoca, 400372 Cluj-Napoca, Romania; dianaadabochynska@gmail.com

**Keywords:** equine herpesvirus, EHV-4, donkey, serology, outbreak

## Abstract

Equine herpesviruses are important pathogens causing significant economic loss in equine and asinine populations. EHV-1/4 strains are mainly associated with respiratory distress. The aim of this study is to report the first EHV 4-associated respiratory disease in donkeys in Romania. Thirty-seven of three hundred jennies in an ecological donkey farm in southwest Romania started initially showing signs of severe upper respiratory tract disease, with ten concomitant late abortions/neonatal deaths and three neurological cases. There were nine fatalities. Pathological examination was performed, and samples were collected for Real-Time PCR analysis and histology. In addition, serum samples from 28 individuals with respiratory symptoms were collected and tested using indirect ELISA. RT-PCR identified the EHV-4 strain. Acute, diffuse necrotizing bronchointerstitial pneumonia with occasional intraepithelial intranuclear viral inclusion bodies was identified. Additionally, EHV-1/4-specific antibodies were found in 15 of the 28 sampled animals. Few studies on donkeys and herpesviruses have been published, and this is the first reported case of EHV-4 outbreak in Romania. There is a need for more extensive seroprevalence studies as, currently, the status of EHV-4 infection in donkeys in Romania is unknown.

## 1. Introduction

Equine herpesvirus infection is a common occurrence in equid populations worldwide. While being the most important, for the Alphaherpesvirinae of the genus Varicellovirus, the reportable EHV1 and EHV4 are only two of the nine so-far identified herpesviruses [1,2]. Herpesvirus infections, while usually confined by the host’s immune system, persist in infected cells, leading to a state of latency [3]; in this case, it leads to latentcy in lymphoid and neural tissue [1] and can lead to viral shedding and the transmission of disease [4]. While EHV-1 is considered a more severe infection, with frequent abortions and neurologic manifestations, EHV-4 has historically been seen as the milder disease, with lesser mortality and predominantly mild respiratory symptoms such as serous nasal discharge that in time becomes mucopurulent [2,5]. The prevalence of EHV-1 and/or 4 among equid populations of all types around the world is high yet not sufficiently investigated in donkeys. It can be up to over 90% in older donkeys [6,7] and the virus circulates subclinically amongst mares and their foals only for symptoms to occur after passive, maternal immunity wanes or when the latent virus is reactivated by periods of stress such as weaning, castration, transport or preparation for sales [5,8,9,10], especially in equines less than one year old [11]. In a recent study, EHV-1 was detected in much higher proportion in donkeys compared to horses [12]. EHV-4 infections with upper respiratory clinical signs tend to be associated with young horses as well [13]. The diagnosis of herpesvirus 1/4 infection and shedding is based on quantitative polymerase chain reaction (qPCR) or real-time (RT) PCR of nasal swabs [4,14,15], but an ELISA serology assay with significant increases in antibodies is also indicative of recent infections [16]. In this paper, we report a severe outbreak of EHV-4 infection with associated abortions, neurological disease and mortality due to environmental and medical stress in an ecological donkey farm in Romania. To the author’s knowledge, this is the first report of a successful isolation of EHV-4 from a donkey population with associated upper respiratory disease in Romania.

## 2. Outbreak Description

### 2.1. Background and Premises

In early April 2021, a total of 37 individuals in an ecological donkey farm in southwest Romania started showing signs of severe respiratory disease after previous introduction (12 days before the first symptoms) of a newly acquired and unquarantined jenny. This farm housed 300 donkeys (290 females and 10 uncastrated males) for milk production as well as two horses for pleasure on various paddocks with full access to the outdoors during both the day and night, with no rigorous separation between paddocks, and one stable is designated for temporary housing. Ages ranged from 0 to 7 years old. None of the animals were vaccinated in the past two years for any infectious disease; some had baseline immunization against tetanus and influenza, but none had immunity for equine herpesvirus. None of them had been dewormed in the past 3 years as well; they had multiple concomitant strongyle infections, and they had not received professional medical care such as teeth or hoof evaluation and treatment.

The affected individuals initially showed serous nasal discharge, which progressed to mucopurulent discharge and in some cases dyspnea and severe apathy, as well as coughing and pyrexia. During the course of two weeks, there were ten fetal losses (caretakers found the fetuses in the morning, there were two losses per night in five consecutive nights, eight were stillborn and in two cases and the foals died several hours after birth) and three neurological cases accompanying the respiratory symptoms. Eight of the affected animals died on the farm in the first ten days of the outbreak, and one was euthanized in a referral clinic due to the severity of the disease. All deceased animals developed severe anorexia and progressed to hyperlipemia rapidly prior to death. After the first 6 days, the owner agreed to impose biosecurity measures on the farm (complete quarantine of sick animals with different caretakers and a different pasture, regular thermometry for all remaining clinically healthy animals and isolation of each if temperature increased and disinfection of the stable where the outbreak started).

### 2.2. Diagnosis

**Figure 1:**
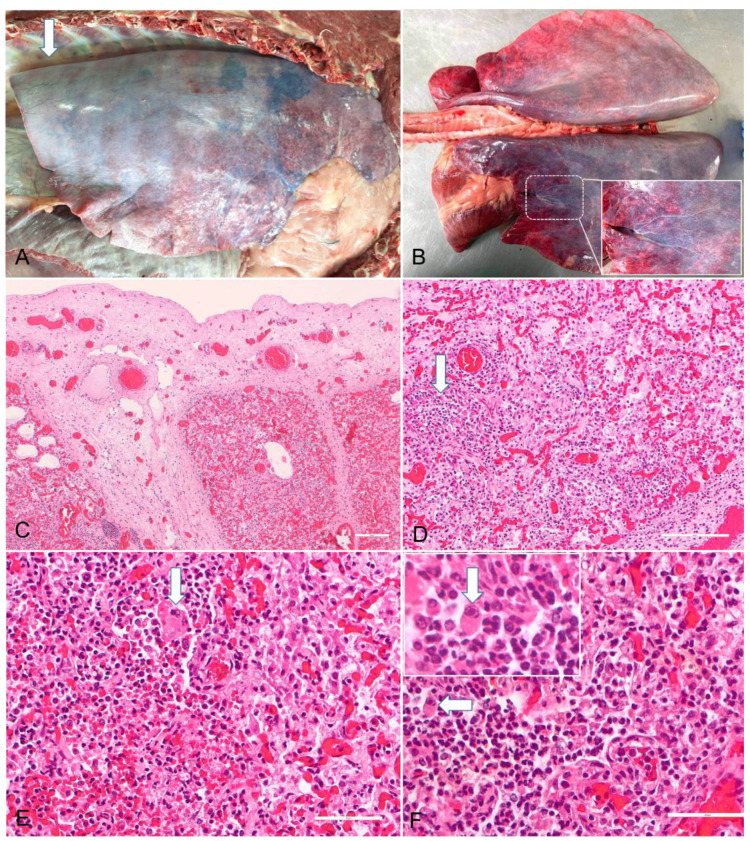
Pathological findings of herpesviral bronchointerstitial pneumonia in a donkey. (**A**) The pleural cavity showing a moderate amount of clear fluid (arrow); (**B**) the lungs failed to collapse after opening the thorax and showed a diffuse rubbery–firm texture with prominent rib imprints, and subpleural and interstitial edema with lymphangiectasia (demarcated area and higher magnification in the inset); (**C**) severe congestion, subpleural and interstitial edema with lymphangiectasia. HE stain, Bar = 50 μm; (**D**) necrotizing and neutrophilic bronchiolitis (arrow). HE stain, Bar = 50 μm; (**E**) epithelial syncytial cells (arrow). HE stain, Bar = 20 μm; (**F**) intranuclear viral inclusion bodies (Cowdry A type) in the bronchiolar epithelium (arrow). HE stain, Bar = 20 μm; inset showing the intranuclear inclusion body at higher magnification. HE stain.

Postmortem examinations on four donkeys were performed. In addition, serum samples from 28 individuals with respiratory symptoms (Table 1) were sent for EHV serology. The tissue samples (lungs bronchial lymph node, nervous system (spinal cord and brain), spleen, heart, kidneys and liver tissues) were collected, as proposed by Wang et al. in 2007 [17] (nasal swab using nylon flocked swabs in polypropylene tubes, Mole Bioscience, China, as well as pulmonary tissue), from the euthanized animals and sent for the molecular detection of EHV1 and EHV4. A standard extraction method was used and an in-house developed Real-Time PCR using the glycoprotein B (gB) gene as the target was performed in a commercial laboratory. PCR identified the EHV-4 strain. In all four cases, postmortem examination revealed approximately 200–500 mL of straw colored, transparent, serous fluid in the thoracic cavity (hydrothorax) with marked subpleural and interlobular edema with foamy fluids within the airways. The lungs failed to collapse when the thorax was opened and showed a diffuse rubbery–firm texture with prominent rib imprints (Figure 1A,B). Pleural and epicardial petechial and echymotic hemorrhages were also found. In two cases, the pericardial sac was moderately distended with serous fluid (hydropericardium). The mediastinal lymph nodes were swollen, edematous and dark red. The spleen and neuroparenchyma were markedly congested. No significant gross changes were observed in other thoracic organs. For histology, lung tissue samples were fixed in 10% neutral buffered formalin for 24 h and embedded in paraffin wax. Three-micrometer-thick sections were stained with hematoxylin and eosin (H&E). Microscopically, necrotizing bronchointerstitial pneumonia and bronchiolitis (Figure 1C,D) with epithelial syncytial cells and occasional eosinophilic intranuclear inclusion bodies of the bronchiolar and alveolar epithelium consistent with herpesviral infection (Figure 1E,F) were identified.

For all 28 jennies upon which serology was performed, samples were also taken for parasitological examination. Rectal feces samples were obtained from each animal, kept at 4 °C in sterile sample cups (60 mL, Prima, Romania) and examined within 24 h. Parasitology examination revealed that 25/28 samples were positive for a moderate to severe burden of strongyles (89.29% prevalence).

**Figure 2:**
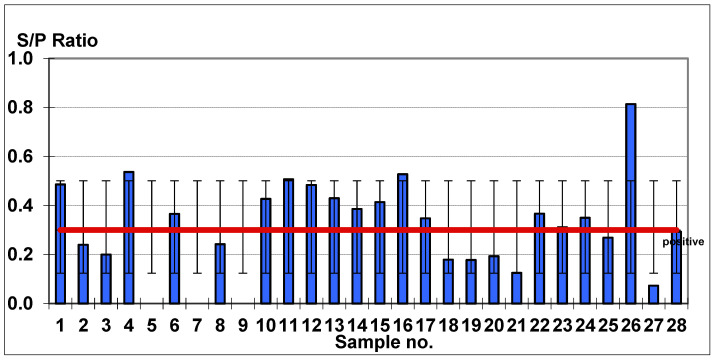
S/P ratios of 0.3 and above were considered cut-off values for positive samples. Fifteen out of twenty-eight serum samples were positive.

Serum samples were collected by venipuncture in specific polyethylene tubes (Vacuette, Greiner Bio-One Austria) three weeks after the initial outbreak, from 28 individuals with URT symptoms, and tested using indirect enzymatic immunoassay (INgezim Rhinopneumonitis EHV1/EHV4, Spain). A serum sample containing specific antibodies against EHV4 binds the antigen adsorbed on plate. After washing, the presence of immunoglobulins was detected using a specific peroxidase conjugate. A colorimetric reaction developed after the substrate addition and was measured by an ELISA reader. The degree of blue color that develops (optical density measured at 405 nm) is directly proportional to the quantity of antibodies present in the sample, compared to that used as a positive standard. As S/P ratio scores depend on different kits based on the manufacturer’s indications; in our case, the cut-off value was 0.3; therefore, the sample was considered positive if S/P ratio score was ≥0.3 and negative if the S/P ratio score was ≤0.3. EHV1/EHV4-specific antibodies were found at relevant S/P ratio scores (i.e., ≥0.3) in 15 of the 28 sampled animals; the positive titers varied from 0.311 S/P to 0.814 (Table 1, Figure 2, Table S1), with a median score of 0.427. Based on symptomatology, morbidity was 12.33% (37/300), mortality was 3% (9/300) and the case fatality rate was 24.32% (9/37).

## 3. Discussion

This study presents the evolution and outcome of a severe outbreak of EHV-4 infection in an ecological donkey farm, with the molecular detection of EHV-4 conducted through Real-Time PCR, histological evaluation and the subsequent determination of EHV1/EHV4 antibodies in the affected animals by using an indirect ELISA method.

While examining the outbreak, the occurrence was observed in donkeys with overt clinical signs and a higher morbidity and mortality than expected for EHV-4, and the main factors that might have led to this are, in our opinion, a combination of the following: the introduction of a jenny with unknown disease status and no quarantine due to a lack of clear protocol in the farm, change of season and housing and chronic lack of adequate medical care. The index case remains unknown for sure, and as EHV-4 can establish latency for life after primary infection with possible reactivation [2], it is possible that reactivation was triggered by different contributing factors. However, the clinically overt cases started 12 days after the introduction of a jenny that was not quarantined, and the probability is high that she was the initial source of the virus. The risk of viral spread in the absence of clinical signs is a possibility in any equid, and proper biosecurity protocols are needed to reduce the risk of introduction, establishment and spread of diseases to, from and within an animal population, and there are official recommendations already available for both horses and farms that can also be applied to a donkey dairy farm [18,19], part of which were implemented in this case as well. Equine rhinopneumonitis transmission occurs mainly by direct contact between hosts when the virus is shed in nasal discharge, which can travel by aerosol conveying infectious particles. There is evidence for aerosol transmission for both EHV-1 and 4 [20,21], which was highly likely in our cases as well. It is unclear whether animals can contract herpesvirus infections from fomites, which likely depends on multiple factors; however, there have been case reports of possible surface contamination and subsequent infection [22]. While equid herpesviruses can also be detected in feces and water, it is still unclear whether transmission can occur through these means [21].

Unfortunately, the lack of a clear protocol at the state level, as well as not having a thorough national animal disease reporting system, renders these types of outbreaks even more dangerous. To this day, there are no OIE WAHIS (World Animal Health Information System) reports on herpesvirus infections in the country. Romania still relies on working equids and donkeys in agriculture and cohabitation with horses is frequent; thus, exposure to viruses can become a stringent problem. According to Pritchard et al., 2005 [23], over 95% of donkey world population can be found in developing countries; however, with the advent of biological farming and increased interest in donkey milk properties, this could result in higher donkey populations in more developed countries where they might represent a medical threat to the local horse population if unmonitored. The latest EFSA panel on EHV infections has concluded that the high seroprevalence of disease in donkeys, as well as the possibility of latent infections might lead to donkeys serving as reservoirs for other equids, but due to lack of data in Europe, the conclusions of the panel did not lead to unanimity in many key domains, such as the impact of the EU economy or animal welfare [24]. Due to uncontrolled animal movement in and out of the area, living conditions and reduced vaccination status, testing and correct biosecurity measures are extremely important for heard health. The socio-economic and cultural status regarding horses in Romania is similar to the situation described by Yildirim et al. 2015 [7] in Turkey; thus, it is possible that seroprevalence of EHV4 is high but yet remains unknown. Several seroprevalence studies in donkeys have shown that this is the case in many countries where donkeys are common (Table 2). The risk of transmission of disease lies not only in the rest of the country but also across the border as Hungary is very close to the location of the outbreak premises. The rapid containment of the outbreak after the initial cases suggests that the biosecurity measures taken were effective and directly induced, rather than incriminating reactivation. The twice-daily temperature measurements, as recommended by the EHV1 consensus statement [25], and the isolation of individuals that showed temperature abnormalities were also efficient.

The affected animals during this outbreak had a median age of 4 years old, in contrast to most of the literature, which sees young animals under the age of one as more susceptible to EHV4 [8,11]. The same study noted a lack of coughing in EHV4 infections but with nasal discharge and fever, submandibular lymphadenopathy and occasionally neurological symptoms. However, most case reports focus on sport horses, and there might be particularities in donkeys, as is frequent for many clinical manifestations of other infectious diseases. All 37 jennies that showed that clinical symptoms were febrile and showed coughing and mucopurulent discharge to a certain degree, and three of them also presented neurological symptomatology such as mild hind limb ataxia and recumbency before death. The ten jennies that lost foals either aborted full term, birthing stillborn foals or, in two cases, the foals were born alive and died several hours later. Males were over-represented in the Pusterla et al. 2011 [11] study, whereas the Romanian population comprised only jennies, due to the specificities of the herd. Pusterla et al. 2011 [11], as well as Badenhorst et al. [8], note that the months with a higher incidence are usually during fall–winter; Matsumura et al. [39] concluded that infections can become clinically overt year-round. Our outbreak took place during the seasonal change to spring, similarly to a very recent paper on EHV4 outbreak in German horses [40]. While most reports [1,2,8] note that EHV-4 infections are usually inapparent and proceed unnoticed, the outbreak in the Romanian farm had a higher morbidity than expected and led to significant economic losses. A paper [8] that analyzed fecal glucocorticoid metabolites in equines as a measurement of stress and correlated it to viral shedding might also explain the appearance of the outbreak in a period of higher stress due to increased parasitism and changes in season. The herd had several weak management strategies such including a non-existing pasture management protocol (no organization when moving from pasture to pasture and no cleaning of pastures between lots of donkeys), lack of organized feeding (competitive feeding) and poor housing (rudimentary stall, porous walls, irregular cleaning of bedding, low ceilings and reduced ventilation), which have possibly led to increased transmission of disease, and preventative care was completely lacking. The sale of organic food items in the EU has doubled in the past years and interest for this type of product is higher than ever [41]. Donkey milk is considered a healthy substitute for breast milk or an alternative to cow’s milk in children with cow’s milk allergy; it has antimicrobial properties and it must respect the same standards as organic cow’s milk [42]. European Union organic farming regulations aim to both offer high standards in animal welfare and to also promote sustainable management in agriculture; however, this can be vague in terms of actual application [43] and, in the case of this donkey farm, might have contributed to a high morbidity and mortality of the outbreak. Moreover, anthelmintic treatment is restricted in dairy production due to the elimination of different molecules in milk, which, in cattle, leads to higher parasite infection rates [44] and might do so as well in milk producing donkeys. Recent studies in ecological donkey farms [45] found multiple parasite infection in almost three quarters of donkeys, which is concerning from both a zoonotic [46] and a welfare point of view. High parasitic burdens induce changes in the immune system [47,48] and might modulate host response and susceptibility to other infectious diseases [49]. While there is no study to date to specifically examine this interaction in farmed donkeys, it is a safe guess to appreciate that the lack of deworming in this population as well as the high parasitic burden of strongyles has contributed to a less efficient immune system and possibly explains the higher morbidity and, especially, case rate fatality than expected in EHV4 infections in this outbreak.

Upper respiratory tract symptoms were apparent in 12.33% of herd members, but true serology status remains unknown due to the unwillingness of the owner to participate, and it is probably much higher. Abortions have rarely appeared in mares infected with EHV-4 [50,51,52]; however, in our case, 24.32% of the jennies with clinical symptoms aborted—either due to the virus itself or due to poor conditions. In the most recent paper on an EHV-4 outbreak [40], the authors have not noted any abortions nor mortality; thus, our outbreak might have involved a different strain or had other external factors that influenced the outcome and might warrant a more in-depth look into genotyping the viral strain.

The entire herd had never been vaccinated against EHV-1/4 and only had one vaccine against Influenza and tetanus in the past two years, which probably contributed to an overall deficient immune response during viral infection. The situation in Romania regarding EHV-4 is quite similar to other countries where owner education is lacking and infectious upper respiratory tract infections are not regarded as a dangerous potential threat to animal health and production [53]. Regular immunization remains questionable for EHV-4 infection prevention, as even in vaccinated individuals, the virus circulates especially between mares and foals [54]; however, repeated vaccination might lessen economical loss within the farm by preventing abortion storms and have the patients present milder clinical symptoms.

While this study analyzed an outbreak of EHV-4 in a donkey herd and reports the first documentation of such an occurrence in Romania, it does have several limitations: (i) there was no possibility of type-specific testing for EHV1/4 antibodies, (ii) only a group with symptoms instead of the entire herd was tested in order to observe real prevalence due to financial constraints of the owner and (iii) sequencing of the virus was not available.

## 4. Conclusions

There are very few studies on donkeys and herpesviruses worldwide, and this is the first reported case of EHV-4 outbreak in Romania. The large increase in donkey population in the country renders the investigation and report of such outbreaks vital. The high morbidity of the clinical disease induced by EHV-4 infection in this ecological donkey farm is an indicator that specific management conditions for these populations might contribute to the expression of more severe clinical disease, with high impacts on the medical and economical status of the herd. There is a need for more extensive seroprevalence studies as, currently, the status of EHV-4 infections in donkeys in Romania is unknown.

## Figures and Tables

**Table 1:** Overview of symptoms and ELISA (S/P cutoff 0.3) results.

Mean Age, Age Ranges of Affected Animals	URT Symptoms	Pyrexia	Abortion	EHV Positive Based on Antibody Titer (S/P > 0.3)	EHV Negative Based on Antibody Titer (S/P < 0.3)
4.2 years (3–6 years)	28/28	28/28	3/28	15/28	13/28

**Table 2:** Overview of reports documenting seroprevalence of EHV-1 and EHV-4 in donkeys, 2008 to present.

Report	EHV Type, Symptomatology	Percentage of Positives	Country, Year	Reference
1	EHV-1	6.6% (1/15)	Brazil, 2008	[26]
2	EHV-1, both symptomatic and asymptomatic	24.2% (31/128)	Turkey, 2009	[19]
3	EHV-1 and 4, asymptomatic	0% (0/4)	Brazil, 2010	[27]
4	EHV-4, asymptomatic	69.7% (134/192)	Bulgaria, 2011	[28]
5	EHV-1 EHV-4 asymptomatic	20.2% (21/104) 84.6% (88/104)	Ethiopia, 2014	[29]
6	EHV-1, asymptomatic	33.3% (4/12)	Tanzania, Namibia, 2015	[30]
7	EHV-1, symptomatic	14.28% (2/14)	EUA, 2016	[31]
8	EHV-1 EHV-4 Asymptomatic	51.85% (126/243) 64.2% (156/243)	Turkey, 2015	[7]
9	EHV-1 and/or 4, asymptomatic	69.5% (57/82)	Sudan, 2016	[32]
10	EHV-4, asymptomatic	14.8% (16/108)	Iran, 2016	[33]
11	EHV-1, symptomatic	98.79% (82/83)	Ethiopia, 2017	[34]
12	EHV-1 and/or 4	74.7% (201/269)	Ethiopia, 2017	[6]
13	EHV-1, symptomatic EHV-4, symptomatic EHV-1, asymptomatic EHV-4, asymptomatic	19.5% (8/41) 9.8% (4/41) 0% (0/10) 0% (0/10)	Ethiopia, 2017	[35]
14	EHV-1 EHV-4 asymptomatic	10% (4/40) 53% (21/40)	West Indies, 2017	[36]
15	EHV-1 and/or 4, asymptomatic	47% (40/85)	Brazil, 2017	[37]
16	EHV-1 EHV-4 asymptomatic	50% (8/16) 6.25% (1/16)	Egypt, 2019	[38]
17	EHV-1, symptomatic	76% (19/25)	Ethiopia, 2021	[12]

## Data Availability

Not applicable.

## Supplementary Materials

The following supporting information can be downloaded at: https://www.mdpi.com/article/10.3390/vaccines10030468/s1, Table S1: Indirect ELISA results for EHV-1/4 anitbodies. y—years; m—months; d—days.
