# Supplementary material for: An Outbreak of Equine Herpesvirus-4 in an Ecological Donkey Milk Farm in Romania
Source: Vaccines (Basel). 2022 Mar 18;10(3):468. doi: 10.3390/vaccines10030468 (PMC8953855; doi:10.3390/vaccines10030468)
Supplement: Supplementary file 1 [file vaccines-10-00468-s001.zip › vaccines-1618324-supplementary.pdf]

vaccines

Case Report

An Outbreak of Equine Herpesvirus-4 in an Ecological Donkey Milk Farm in Romania

Alexandra Mureşan [1,*], Cosmin Mureşan [2], Madalina Siteavu [3,4], Electra Avram [3], Diana Bochynska [5] and Marian Taulescu [3,5]

Table S1. Indirect ELISA was performed on serum samples in all surviving animals that showed URT symptoms and/or abortion and/or pyrexia. Animal demographics and data on antibody testing as well as results were interpreted. Y—years, M—months, D—days.

Nr.	Species	Age	Sex	Symptoms	Antibody titer (S/P)	Result
1.	Donkey	5 Y	F	URT symptoms, pyrexia	0.486	Positive
2.	Donkey	10 M	F	URT symptoms, pyrexia	0.240	Negative
3.	Donkey	4 Y	F	URT symptoms, pyrexia	0.200	Negative
4.	Donkey	4 Y	F	URT symptoms, pyrexia	0.537	Positive
5.	Donkey	7 Y	F	URT symptoms, pyrexia	0.000	Negative
6.	Donkey	6 Y	F	Abortion, URT symptoms, pyrexia	0.366	Positive
7.	Donkey	7 D	F	URT symptoms, pyrexia	0.000	Negative
8.	Donkey	5 Y	F	URT symptoms, pyrexia	0.242	Negative
9.	Donkey	10 D	F	URT symptoms, pyrexia	0.000	Negative
10.	Donkey	5 Y	F	URT symptoms, pyrexia	0.427	Positive
11.	Donkey	3 Y	F	URT symptoms, pyrexia	0.507	Positive
12.	Donkey	3 Y	F	URT symptoms, pyrexia	0.484	Positive
13.	Donkey	4 Y	F	URT symptoms, pyrexia	0.430	Positive
14.	Donkey	4 Y	F	URT symptoms, pyrexia	0.386	Positive
15.	Donkey	4 Y	F	URT symptoms, pyrexia	0.414	Positive
16.	Donkey	5 Y	F	Abortion, URT symptoms, pyrexia	0.528	Positive
17.	Donkey	6 Y	F	URT symptoms, pyrexia	0.348	Positive
18.	Donkey	6 Y	F	URT symptoms, pyrexia	0.179	Negative
19.	Donkey	5 Y	F	URT symptoms, pyrexia	0.178	Negative
20.	Donkey	6 Y	F	Abortion, pyrexia	0.193	Negative
21.	Donkey	5 Y	F	URT symptoms, pyrexia	0.125	Negative
22.	Donkey	3 Y	F	URT symptoms, pyrexia	0.367	Positive
23.	Donkey	4 Y	F	URT symptoms, pyrexia	0.311	Positive
24.	Donkey	5 Y	F	URT symptoms, pyrexia	0.350	Positive
25.	Donkey	6 Y	F	URT symptoms, pyrexia	0.269	Negative
26.	Donkey	3 Y	F	URT symptoms, pyrexia	0.814	Positive
27.	Donkey	7 Y	F	URT symptoms, pyrexia	0.073	Negative
28.	Donkey	6 Y	F	URT symptoms, pyrexia	0.293	Negative
